# Combined Treatment of Hydroxytyrosol with Carbon Monoxide-Releasing Molecule-2 Prevents TNF****α****-Induced Vascular Endothelial Cell Dysfunction through NO Production with Subsequent NF****κ****B Inactivation

**DOI:** 10.1155/2013/912431

**Published:** 2013-08-27

**Authors:** Houda Zrelli, Che Wei Wu, Nahla Zghonda, Hidehisa Shimizu, Hitoshi Miyazaki

**Affiliations:** ^1^Faculty of Life and Environment Sciences, University of Tsukuba, 1-1-1 Tennodai, Tsukuba, Ibaraki 305-8572, Japan; ^2^Research Faculty of Agriculture, Hokkaido University, Kita 9, Nishi 9, Kita-ku, Sapporo, Hokkaido 060-0808, Japan

## Abstract

This study investigated the atheroprotective properties of olive oil polyphenol, hydroxytyrosol (HT), in combination with carbon monoxide-releasing molecule-2 (CORM-2) that acts as a carbon monoxide donor using vascular endothelial cells (VECs). Our results showed that CORM-2 could strengthen the cytoprotective and anti-apoptotic effects of HT against TNF**α**-induced cellular damage by enhancing cell survival and the suppression of caspase-3 activation. While HT alone attenuated NF**κ**Bp65 phosphorylation and I**κ**B**α** degradation triggered by TNF**α** in a dose-dependent manner, combined treatment of HT with CORM-2 but not iCORM-2 nearly completely blocked these TNF**α** effects. Furthermore, combined action of both compounds results in the inhibition of NF**κ**B nuclear translocation. Results also indicate that both compounds time-dependently increased eNOS phosphorylation levels and the combination of HT with CORM-2 was more effective in enhancing eNOS activation and NO production in VECs. The NOS inhibitor, L-NMMA, significantly suppressed the combined effects of HT and CORM-2 on TNF**α**-triggered NF**κ**Bp65 and I**κ**B**α** phosphorylation as well as decreased cell viability. Together, these data suggest that carbon monoxide-dependent regulation of NO production by the combination of HT with CORM-2 may provide a therapeutic benefit in the treatment of endothelial dysfunction and atherosclerosis.

## 1. Introduction

Endothelial dysfunction is considered an earlier marker for the initiation and progression of atherosclerosis and a predictor of future clinical cardiovascular events [1, 2]. Therefore, the early detection and treatment of endothelial dysfunction is of great importance for combating cardiovascular complications. Various factors are implicated in the initial injury of vascular endothelium and the prolonged inflammation of the vasculature. Among them, reactive oxygen species (ROS), proinflammatory cytokines such as tumor necrosis factor *α* (TNF*α*), and impaired nitric oxide (NO) bioavailability are thought to be critically involved [3–5]. 

**Figure 1:**
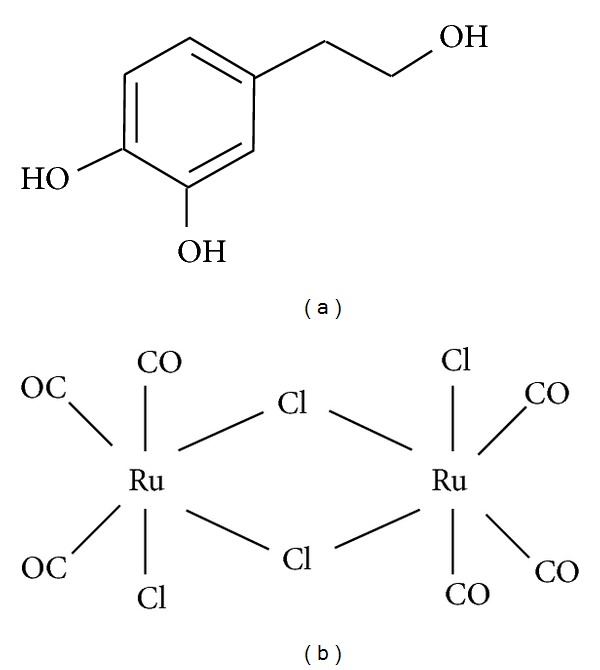
Chemical structure of (a) Hydroxytyrosol: C_8_H_10_O_3_ and (b) CORM-2: [Ru(CO)_3_C_l2_]_2_.

There is a growing interest in discovering strategies to inhibit endothelial injury leading to increased endothelial cell apoptosis. The lower rates of cardiovascular disease and certain cancers in the Mediterranean regions have been attributed to the dietary habits of the people in that area [6]. One of the most characteristic elements of the Mediterranean diet is undoubtedly olive oil consumption. Hydroxytyrosol (HT) (Figure 1(a)) is a polyphenol found in olive oil and leaves. Past studies showed that HT exhibits protective effects on various diseases including cardiovascular disease, which has been attributed to its antioxidative and anti-inflammatory activities [7–9]. In fact, HT has been shown to halt the progression of atherosclerotic lesions formation through its antioxidant capacity, especially against lipid peroxidation and LDL oxidation both in human and animal [10, 11]. Other studies have indicated that HT reduces endothelial adhesion molecule expressions as well as the expression of proinflammatory and proatherogenic genes [12, 13]. It has also been demonstrated that HT protects the vascular tone against oxidative stress-induced impairment in NO-mediated relaxation by quenching vascular intracellular free radicals [14]. Moreover, we have recently found that HT positively regulates the antioxidant defense system involving heme oxygenase-1 (HO-1) and catalase to inhibit oxidative vascular damage and promote wound healing of porcine vascular endothelial cells (VECs) [15, 16].

Carbon monoxide (CO) is endogenously produced as a by-product in the catabolism of heme by HO-1 and actively participates in the regulation of intracellular events as a key factor. Recently, transitional metal carbonyls, termed CO-releasing molecules (CORMs), were used in biological systems to provide better approach for the delivery of CO in a safe and controlled way [17]. Emerging evidence reveals that CO plays a critical role in the resolution of inflammatory processes and alleviation of cardiovascular disorders [18–20]. CO has been shown to relax blood vessels and act as a vasodilator on aortic and cardiac tissues of rats [21]. The exogenous addition of CO exerted potential anti-inflammatory effects on endothelial cells and mediated cytoprotective effects mimicking those of HO-1. CORM-3 reduced inflammatory responses both *in vitro* and *in vivo* [22, 23]. In addition, CO administration as CORM-A1 inhibited NADPH oxidase activation by TNF*α* and reduced oxidative stress-induced apoptosis, resulting in the prevention of cerebral vascular endothelial cells injury [24].

In the present study, we investigated the combined effects of HT and CORM-2 (Figure 1(b)) using VECs. Treatment with a combination of these two compounds showed an additional benefit on cell survival and cytoprotection against TNF*α*-induced cell damage. We also explored their underlying molecular mechanisms.

## 2. Materials and Methods

### 2.1. Reagents, Chemicals, and Antibodies

Dulbecco's modified Eagle's medium (DMEM), fetal bovine serum (FBS), penicillin and streptomycin, Hanks' balanced salt solution (HBSS), dimethyl sulfoxide (DMSO), L-arginine, and tricarbonyldichlororuthenium (II) dimer [Ru(CO)_3_C_l2_]_2_, CORM-2) were all purchased from Sigma Chemical Co. (St. Louis, MO, USA). Hydroxytyrosol (3,4-dihydroxyphenyl ethanol) was purchased from Cayman Chemical Co. (Ann Arbor, MI, USA). 4,5-Diaminofluorescein diacetate (DAF-2 DA) was obtained from Daiichi Pure Chemicals Co. NG-monomethyl-L-arginine, monoacetate salt (L-NMMA), and anti*α*-tubulin antibody were from Calbiochem. NF*κ*B pathway sampler kit, antibody specific for phospho-eNOS, anticleaved caspase-3, and horseradish peroxidase-linked antirabbit IgG were obtained from Cell Signaling Technology, Inc. (Beverly, MA, USA). Antimouse IgG, horseradish peroxidase-conjugated antigoat IgG, and antilamin B were from Santa Cruz Biotechnology, Inc. (Santa Cruz, CA, USA). Recombinant human TNF*α* was purchased from Millipore (Billerica, MA, USA).

### 2.2. Cell Culture

VECs were isolated from porcine pulmonary arteries as previously described [16]. Cells were grown in DMEM containing low glucose (1000 mg/L) supplemented with 10% FBS, 100 U/mL penicillin, and 100 *μ*g/mL streptomycin at 37°C in humidified 5% CO_2_ incubator and experiments were conducted on cells at approximately 80 to 90% confluence. The culture medium was replaced by DMEM containing 1% FBS for 16 h (starvation). Thereafter, cells were treated with HT, TNF*α*, L-NMMA, CORM-2, or inactivated CORM-2 (iCORM-2). iCORM-2 was prepared by incubating the compound in culture medium at 37°C for 24 h in humidified 5% CO_2_ incubator to liberate CO. iCORM-2 solution was also bubbled with nitrogen to remove the residual CO present in the solution.

### 2.3. Preparation of Whole Cell Extracts and Isolation of Cellular Fractions

To prepare whole cell extracts, VECs washed twice with ice-cold PBS were lysed with cell lysis buffer (50% glycerol, 100 mM NaF, 10 mM sodium pyrophosphate, 1% Triton X-100, 1 mM Na_3_VO_4_, 1 mM PMSF, 10 *μ*g/mL antipain, 10 *μ*g/mL leupeptin, and 10 *μ*g/mL aprotinin). The lysates were centrifuged at 15,000 g for 20 min at 4°C and supernatants were collected. Nuclear and cytosolic fractions were isolated as previously described [16]. Briefly, VECs were lysed on ice with lysis buffer (10 mM HEPES (pH 8.0), 10 mM KCl, 0.5 mM DTT, 0.5 mM PMSF, 0.1% Nonidet P-40, 10 *μ*g/mL antipain, 10 *μ*g/mL leupeptin, and 10 *μ*g/mL aprotinin). To separate the cytosolic fractions, cell lysates were centrifuged at 15,000 g at 4°C for 3 min and supernatants were collected. The pellets were resuspended and incubated for 10 min on ice in cold extraction buffer (10 mM HEPES (pH 8.0), 400 mM NaCl, 1 mM EDTA, 1 mM DTT, 10 *μ*g/mL antipain, 10 *μ*g/mL leupeptin, and 10 *μ*g/mL aprotinin). After centrifugation at 15,000 g for 20 min, nuclear fractions were collected. The protein concentration in the extracts was measured using the BCA protein assay kit (Pierce, Rockford, IL, USA).

### 2.4. Western Blot Analysis

Cell extracts containing equal quantities of proteins (30 *μ*g) were electrophoresed in 10% polyacrylamide gel, and proteins were transferred to PVDF membrane using Semi-Dry Transfer Cell (Trans-blot, Bio-Rad, USA). Membranes were blocked with 5% nonfat milk in 10 mM Tris-HCl (pH 7.4), 150 mM NaCl, and 0.05% Tween 20 (TBS-T) for 1 h at room temperature then incubated with appropriate dilutions of primary antibodies against eNOS, NF*κ*Bp65, I*κ*B*α*, cleaved caspase-3, or *α*-tubulin overnight at 4°C. The primary antibodies were then detected using horseradish peroxidase-conjugated donkey antirabbit or goat antimouse secondary antibodies (1/5000). Immunocomplexes were visualized using the Western Lightning Chemiluminescence kit according to the manufacturer's instructions (Santa Cruz, CA, USA). Anti-*α*-tubulin and anti-lamin B antibodies were used to normalize the protein input and to control fraction separation.

### 2.5. NO Production

The fluorometric detection of NO production was assessed using DAF-2 DA, a cell-permeable, and NO-sensitive fluorescent dye as previously described [25]. Briefly, VECs (4 × 10^3^ cells/well) seeded into 96-well plates were incubated with 10 *μ*M DAF-2 DA in the dark for 30 min at 37°C. After washing with HBSS, NO production was stimulated by the addition of HBSS containing 100 *μ*M L-arginine in the presence of the necessary treatment as indicated in the figure legends. Fluorescence was next measured by spectrofluorophotometer (Powerscan HT, Dainippon Pharmaceutical) with excitation and emission wavelengths of 488 and 530 nm, respectively.

### 2.6. Cell Viability and Morphology

Serum-starved VECs were seeded into 96-well plates at the density of 3 × 10^4^ cells/well and incubated with TNF*α* or subjected to the required treatment as described in the figure legend. Cell viability was determined by colorimetric MTS assays kit (Promega), according to the manufacturer's instructions and as previously reported [15]. Observation of cellular morphological changes in VECs after 24 h treatment with TNF*α*, HT, CORM-2, or iCORM-2 was performed using phase contrast inverted microscope at 24 h (Leica, Wetzlar, Germany). The number of dead cells was measured by counting Trypan Blue staining cells using the Countess automated cell counter (Invitrogen).

### 2.7. Statistical Analysis

Data are expressed as means ± S.D. from at least three different experiments. Differences between groups were analyzed by using Student's *t*-test. Values of *P* < 0.05 were considered statistically significant.

## 3. Results

### 3.1. Enhanced Cytoprotective and Antiapoptotic Effects by the Combination of HT with CORM-2 in VECs

**Figure 2:**
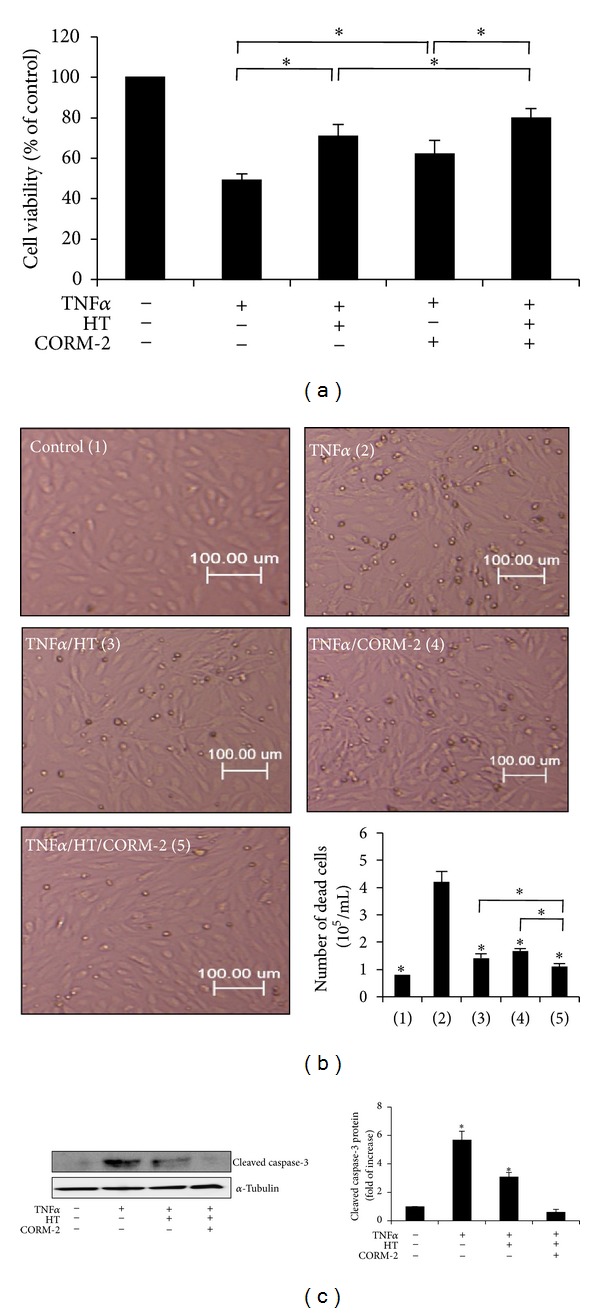
Combined effect of HT and CORM-2 on VECs survival and apoptosis. (a) Serum-starved VECs were treated with 10 ng/mL TNF*α* in the presence or absence of 50 *μ*M HT or CORM-2 or both for 24 h. Cell viability was determined by MTS assay. Data represent the mean ± SD of three different independent experiments (*P < 0.05). (b) Morphological changes characteristic of apoptotic cell death were observed under the phase-contrast inverted microscope, and the total number of dead cells was counted by trypan blue exclusion. Data represent the mean ± SD of three different independent experiments *P < 0.05 versus TNF*α*-treated cells. (c) Serum-starved VECs were treated with 10 ng/mL TNF*α* in the presence or absence of 50 *μ*M HT or 50 *μ*M HT plus 50 *μ*M CORM-2 for 3 h. Cleaved caspase-3 protein expression was determined using western blotting and normalized to the amount of *α*-tubulin. Representative data are shown. Data are the mean ± SD of three independent experiments. *P < 0.05 versus untreated cells.

In order to investigate the cytoprotective properties of HT, CORM-2, and a potential combined effect between these compounds, we induced apoptosis with TNF*α* since it has been shown to trigger apoptosis in various cell types including VECs. As measured by MTS assay (Figure 2(a)), incubation of the cells with 10 ng/mL TNF*α* for 24 h decreased the percentage of cell viability approximately by 50% compared to control cells, while treatment with 50 *μ*M HT or 50 *μ*M CORM-2 obviously prevented TNF*α*-induced cell death, respectively. Combined treatment of HT with CORM-2 significantly enhanced cell protection against TNF*α*-mediated cellular damage. 

Typical apoptotic morphological changes characterized by loss of adherence, condensed cytoplasm, and formation of apoptotic bodies were observed when VECs were exposed to TNF*α*. These changes were decreased in the presence of HT or CORM-2 and were inhibited almost totally by the combination of HT with CORM-2. We also determined the antiapoptotic effect of HT and CORM-2 on VECs in terms of cell death using the trypan blue dye exclusion assay (Figure 2(b)). Treatment of cells with TNF*α* for 24 h resulted in a 5.3-fold increase in cell death compared to control cells, while treatment with HT significantly decreased apoptotic cells number (1.8-fold versus control cells). Similar results were obtained after treatment with CORM-2 (2.3-fold versus control cells). The addition of HT together with CORM-2 enhanced the decrease in the number of dead cells (1.3-fold versus control cells) (Figure 2(b)). In accordance with these data, the level of cleaved caspase-3, a prominent signaling molecule in the apoptosis pathway, was significantly higher in TNF*α*-treated cells compared to control cells. The activation of caspase-3 was completely blocked by the simultaneous addition of HT and CORM-2 (Figure 2(c)). Together, these results demonstrate that combined treatment with HT and CORM-2 has stronger cytoprotective and antiapoptotic effects compared to their individual effect in VECs.

### 3.2. HT Dose-Dependently Inhibits TNF*α*-Mediated NF*κ*B Activation

**Figure 3:**
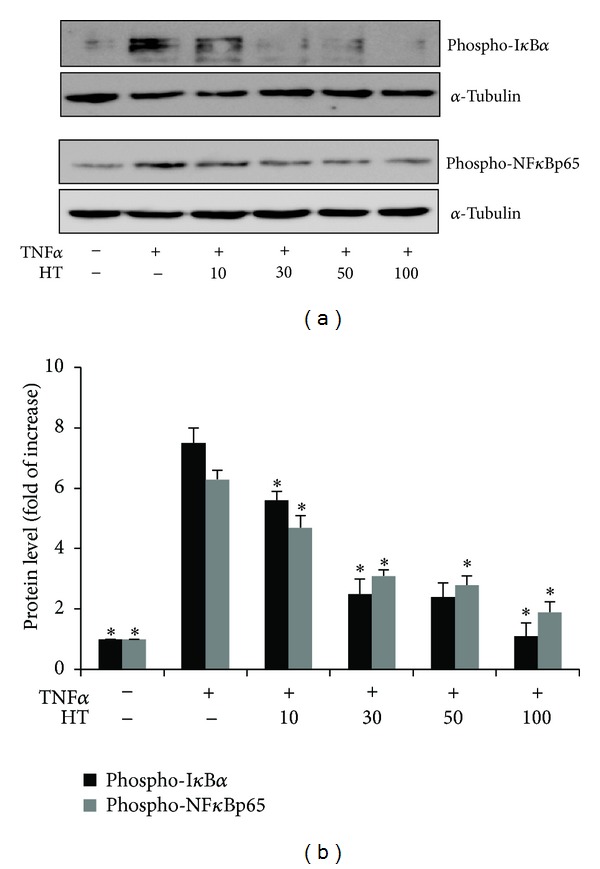
Effect of HT on TNF*α*-mediated NF*κ*B activation. Serum-starved VECs were incubated with 2 ng/mL TNF*α* and HT at different concentrations (10–100 *μ*M) for 3 h. Phospho-I*κ*B*α* (Ser^32^) and phospho-NF*κ*Bp65 (Ser^536^) protein levels were determined using western blotting and normalized to the amount of *α*-tubulin. Representative data is shown from three independent experiments that yielded similar results. *P < 0.05 versus TNF*α*-treated cells.

To know the molecular mechanism underlying the cytoprotective and antiapoptotic properties of HT and CORM-2, further experiments were performed with regard to NF*κ*B activation in VECs. We examined the effect of HT on TNF*α*-induced NF*κ*B activation. Cells were treated with various concentrations of HT (10–100 *μ*M) for 3 h before stimulation with TNF*α* for another 3 h. Western blot data revealed that TNF*α* induced I*κ*B*α* phosphorylation at Ser^32^ which is prerequisite to induce I*κ*B*α* degradation, and also, the phosphorylation of nuclear NF*κ*Bp65 at Ser^536^, required for NF*κ*B activation (Figure 3) [26, 27]. HT effectively blocked TNF*α*-induced I*κ*B*α* and NF*κ*Bp65 phosphorylation in a dose-dependent manner, suggesting that HT inhibits TNF*α*-mediated NF*κ*B activation in VECs.

### 3.3. HT-Induced Suppression of TNF*α*-Mediated NF*κ*B Activation Is Enhanced by CORM-2

**Figure 4:**
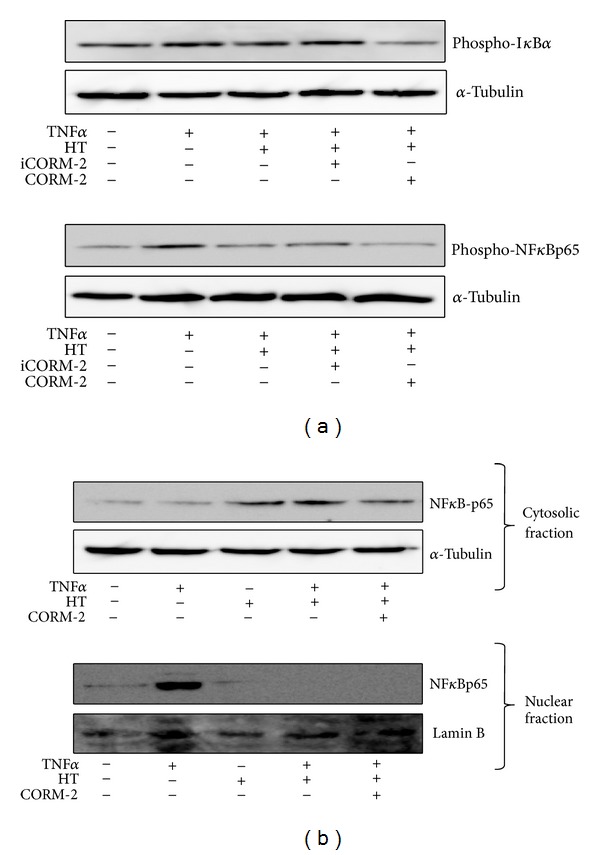
Combined effect of HT and CORM-2 on TNF*α*-mediated NF*κ*B activation. (a) Serum-starved VECs were treated with 50 *μ*M iCORM-2 or 50 *μ*M CORM-2 for 1 h in the presence of HT prior to the addition of TNF*α* (2 ng/mL) for 3 h. Phospho-NF*κ*Bp65 (Ser^536^) and phospho-I*κ*B*α* (Ser^32^) protein levels were determined using western blotting and normalized to the amount of *α*-tubulin. Representative data is shown from three independent experiments that yielded similar results. (b) Serum-starved VECs were treated with HT and CORM-2 (50 *μ*M) for 1 h followed by TNF*α* (2 ng/mL) stimulation for 3 h. NF*κ*Bp65 expression was determined using western blotting in both cytosolic and nuclear fractions. Cytosolic and nuclear fractions were blotted for *α*-tubulin and Lamin B, respectively, for the protein normalization. Representative data is shown from three independent experiments that yielded similar results.

To investigate whether CORM-2 enhances HT induced inhibition of TNF*α*-mediated NF*κ*B activation, we performed western blotting to measure the phosphorylation levels of I*κ*B*α* and NF*κ*Bp65. As expected we found that HT inhibited the effect of TNF*α* on I*κ*B*α* and NF*κ*Bp65 phosphorylation (Figure 4(a)). The combination of HT and CORM-2 almost completely blocked TNF*α*-mediated I*κ*B*α* and NF*κ*Bp65 phosphorylation. In contrast, treatment of VECs with the inactive compound iCORM-2 did not influence these phosphorylation levels (Figure 4(a)). 

We next determined the combined effect of CORM-2 and HT on NF*κ*B protein levels in the cytosolic and nuclear fractions. As shown in Figure 4(b), stimulation of VECs with TNF*α* induced an increase in NF*κ*Bp65 protein level in the nucleus, while this protein level decreased in the cytosol. On the other hand, HT treatment obviously inhibited TNF*α*-mediated nuclear localization of NF*κ*Bp65. Treatment of cells with HT in the presence of CORM-2 also displayed a remarkable decrease in NF*κ*Bp65 protein levels and translocalization. Together, these results demonstrate that TNF*α*-induced NF*κ*B activation was totally blocked by combined treatment of HT with CORM-2.

### 3.4. HT in Combination with CORM-2 Increases eNOS Activation and NO Production in VECs

**Figure 5:**
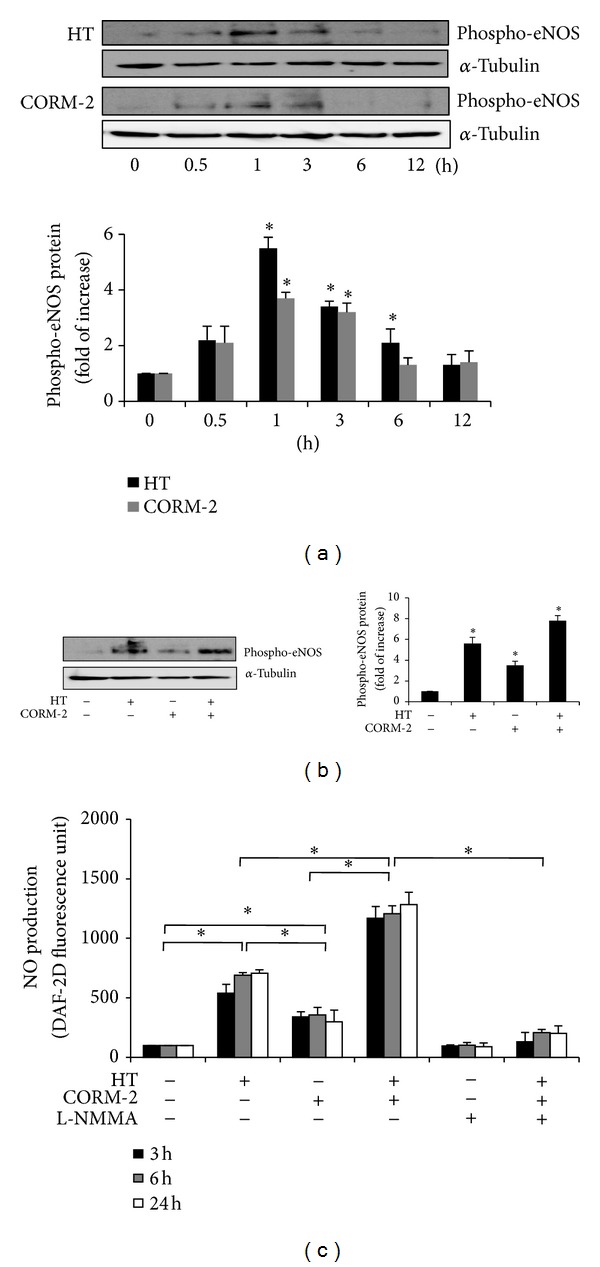
Combined effects of HT and CORM-2 on eNOS phosphorylation and NO production in VECs. (a) Serum-starved VECs were incubated with 50 *μ*M HT or 50 *μ*M CORM-2 for the indicated periods (0 to 12 h). Phospho-eNOS (Ser^1177^) protein levels were determined using western blotting and normalized to the amount of *α*-tubulin. Representative data is shown from three independent experiments that yielded similar results. *P < 0.05 versus untreated cells. (b) Serum-starved VECs were treated with 50 *μ*M HT in the presence of 50 *μ*M CORM-2 for 1 h. Phospho-eNOS (Ser^1177^) protein levels were determined using western blotting and normalized to the amount of *α*-tubulin. Representative data is shown from three independent experiments that yielded similar results. *P < 0.05 versus untreated cells. (c) Serum-starved VECs were treated with 50 *μ*M HT or 50 *μ*M CORM-2 in the presence or absence of 1 mM L-NMMA for 3, 6, and 24 h. NO production was assessed using the fluorescent reagent DAF-2 DA and expressed as a percentage of the control. Data represent the mean ± SD of three different independent experiments (*P < 0.05).

We investigated the effect of HT in combination with CORM-2 on eNOS phosphorylation levels and NO production in VECs, since numerous studies have shown that NO elicits cytoprotective and atheroprotective responses in the vasculature [28–31]. VECs were incubated with 50 *μ*M HT or 50 *μ*M CORM-2 at various periods, and eNOS phosphorylation was examined by western blot. Results showed a time-dependent increase in the protein levels of activated eNOS (phospho-eNOS Ser^1177^) in both HT and CORM-2 treated cells as compared with untreated cells (Figure 5(a)). The combined action of both compounds resulted in a significant enhancement of eNOS phosphorylation compared with HT or CORM-2 treated cells (Figure 5(b)).

 To confirm the biological importance of this increased eNOS phosphorylation, we examined intracellular NO production using vital staining with the fluorescent NO indicator DAF-2. Results revealed that there was a significant increase in DAF-2 fluorescence in cells treated with either HT or CORM-2 as compared with control cells, indicating that intracellular NO production was increased in these cells (Figure 5(c)). The combination of HT and CORM-2 led to a further increase in NO bioavailability compared with cells treated with each compound alone. The NOS inhibitor, L-NMMA, was added to determine whether this NO increase was obtained from NOS derived de novo synthesis. Pretreatment of cells with 1 mM L-NMMA obviously inhibited NO production induced by the combined treatment of HT and CORM-2. The aforementioned data show that HT and CORM-2 act together to promote eNOS activation with subsequent NO production in VECs.

### 3.5. HT in Combination with CORM-2 Increases Cell Survival by Suppressing TNF*α*-Mediated NF*κ*B Activation through NO Production

**Figure 6:**
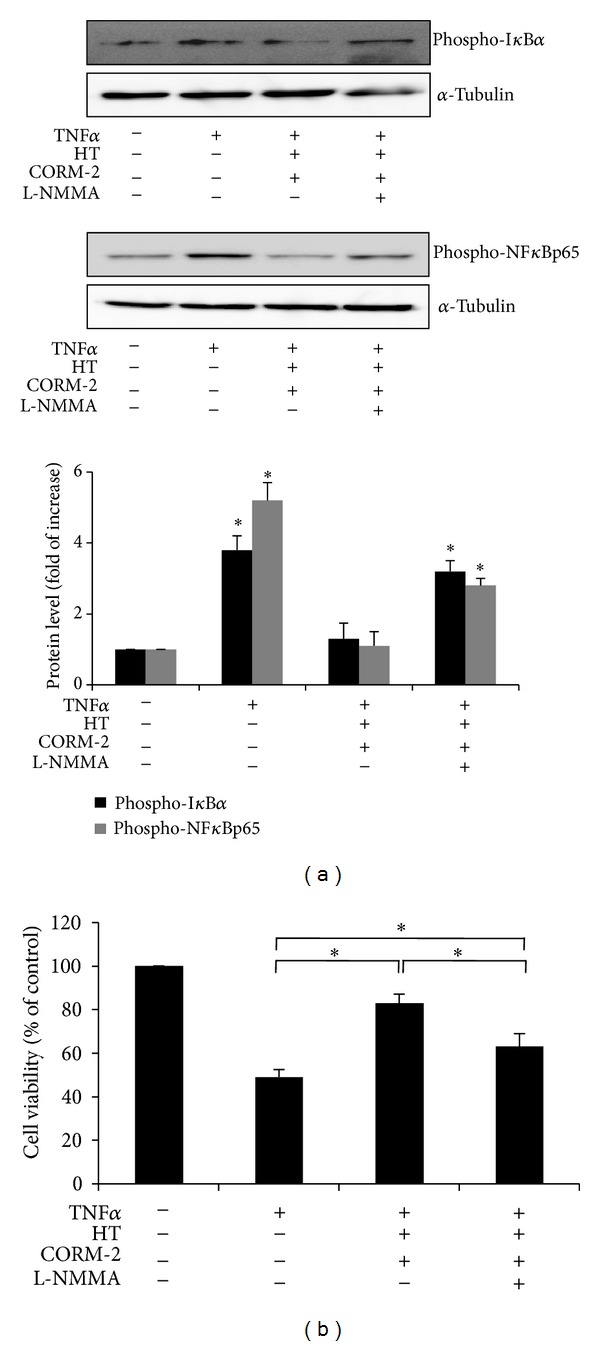
Effect of combined HT and CORM-2-induced NO production on NF*κ*B activation and cell survival. (a) Serum-starved VECs were treated with 1 mM L-NMMA for 1 h prior to the addition of 50 *μ*M HT and 50 *μ*M CORM-2 in the presence of 2 ng/mL TNF*α* for 3 h. Phospho-I*κ*B*α* (Ser^32^) and phospho-NF*κ*Bp65 (Ser^536^) protein levels were determined using western blotting and normalized to the amount of *α*-tubulin. Representative data is shown from three independent experiments that yielded similar results. *P < 0.05 versus untreated cells. (b) Serum-starved VECs were pretreated with 1 mM L-NMMA for 1 h then incubated with 50 *μ*M HT and 50 *μ*M CORM-2 in the presence of TNF*α* (10 ng/mL) for 24 h. Cell viability was determined by MTS assay. Data represent the mean ± SD of three different independent experiments (*P < 0.05).

To elucidate the potential role of NO on NF*κ*B activation and its significance on cell survival, the phosphorylation of I*κ*B*α* and NF*κ*Bp65 triggered by TNF*α* stimulation was measured after treatment of VECs with HT combined with CORM-2 in the presence or absence of L-NMMA. As shown in Figure 6(a), the presence of L-NMMA reversed the reduction in I*κ*B*α* and NF*κ*Bp65 phosphorylation obtained after treatment with HT and CORM-2. In the same way, we evaluated the functional consequence of NO depletion with L-NMMA on VECs growth and survival. As determined by MTS assay, the increase in cell viability induced by HT in combination with CORM-2 was significantly attenuated by L-NMMA (Figure 6(b)). These data clearly demonstrate that NO is involved in the combined action of HT and CORM-2 on regulating NF*κ*B activation and increasing cell survival.

## 4. Discussion

Previous reports have indicated that both HT and CO-releasing molecules (CORMs) exhibit cardioprotective effects through multiple mechanisms of action. The present study took the approach of the combined treatment with HT and CORM-2 to investigate their potential cytoprotective effects on VECs. To the best of our knowledge, this is the first report demonstrating that HT in combination with CORM-2 effectively enhances cytoprotection against TNF*α*-dependent VECs damage compared with those induced by single compounds through the suppression of caspase-3 activation, a common mediator of apoptosis. This combined treatment also inhibited TNF*α*-induced I*κ*B*α* and NF*κ*Bp65 phosphorylation, thereby leading to the suppression of nuclear NF*κ*B activation. Furthermore, both compounds increase eNOS activation and NO production in VECs, which contributes to NF*κ*B inactivation.

Vasoactive peptides such as platelet-derived growth factor and angiotensin II are known to produce H_2_O_2_ in vascular smooth muscle cells which causes VECs damage. Therefore, compounds that can protect VECs from H_2_O_2_-dependent damage are beneficial to prevent atherosclerosis. Our previous studies demonstrated that HT induces the proliferation of VECs and protects the cells against H_2_O_2_ damage by the stimulation of Nrf2/HO-1 pathway, and raising catalase expression, which appears to be crucial to prevent early endothelial dysfunction [15, 16]. The proinflammatory cytokine, TNF*α*, which could be secreted by various cells including smooth muscle cells, macrophages, and adipocytes is also one of the critical factors involved in atherogenesis. The present study demonstrated that HT also protects against TNF*α*-caused VECs damage and that the combination of HT with CORM-2 considerably enhanced cell protection against TNF*α*-mediated cellular damage and promoted VECs survival.

Recently, CORMs that possess the potential to facilitate the pharmaceutical use of CO both *in vitro* and *in vivo* have been shown to play a crucial role to mediate potent anti-inflammatory effects and elicit cardioprotective properties [32, 33]. For example, CORM-A1 was demonstrated to inhibit Nox4 NADPH oxidase activation and prevent the development of oxidative stress-induced apoptosis in TNF*α*-challenged cerebral microvascular endothelial cells [24]. Similarly, it has been described that CORM-2 elicits anti-inflammatory effects by interfering with redox-sensitive cell signaling and inhibition of proadhesive phenotype by interfering with NF*κ*B activation in human umbilical VECs [34]. 

Accumulating evidence suggests that NF*κ*B is critically implicated in various inflammatory diseases. In quiescent cells, inactive NF*κ*B proteins are sequestered in the cytoplasmic compartment associated with members of the I*κ*B family. Upon stimulation with proinflammatory cytokines including TNF*α*, I*κ*B*α* undergoes phosphorylation at Ser^32^ and Ser^36^, which leads to its ubiquitination and proteasomal degradation, resulting in the release of NF*κ*B. A key step in NF*κ*B activation is the phosphorylation of NF*κ*Bp65 subunit at Ser^536^. Phosphorylated NF*κ*B translocates to the nucleus where it binds to the promoter region of multiple genes involved in inflammation [26, 27]. The present study shows that HT inhibits TNF*α*-induced phosphorylation of both I*κ*B*α* and NF*κ*Bp65 as well as the nuclear translocation of NF*κ*B and that the simultaneous stimulation with HT and CORM-2 effectively enhances these responses. In agreement with our data, Carluccio et al. demonstrated that HT reduces adhesion molecule expression and endothelial activation induced by LPS through the inhibition of NF*κ*B and AP-1 activation [35]. In addition, CORM-2 was demonstrated to be associated with the decrease in NF*κ*B activation after LPS stimulation and subsequently attenuated ICAM-1 cell surface levels both *in vivo* and *in vitro* [36]. The subsequent results of NF*κ*B inactivation on the expression of proinflammatory molecules like adhesion molecules following HT and CORM-2 combined treatment will be the focus of our future study.

A number of reports suggest that NO synthesized by eNOS displays a potent role in the prevention of atherosclerosis by regulating vascular tone and maintaining vascular integrity. The present study indicates that NO plays a critical role in the modulation of NF*κ*B activation, and HT and CORM-2 are crucially responsible for this NO-NF*κ*B signaling pathway. Different mechanisms have been proposed to underlie inhibition of NF*κ*B activation by NO. Grumbach et al. showed that NO attenuates shear stress-dependent NF*κ*B activation through *S*-nitrosylation of the NF*κ*B subunit in bovine aortic endothelial cells [37]. Other findings indicate that NO inhibits NF*κ*B activation and VCAM-1 expression by increasing the expression and nuclear translocation of IkB*α* following TNF*α* stimulation in human VECs [38]. Shen et al. also revealed that NO donor *S*-nitroso-*N*-acetylpenicillamine (SNAP) inhibits TNF*α*-induced endothelial apoptosis and this SNAP effect is mediated partly through the cGMP pathway [39].

In summary, this is the first study to show that the combination of HT with CORM-2 exhibits an enhanced effect on growth and survival of VECs. This combination also has additional cytoprotective and antiapoptotic properties by inhibiting NF*κ*B activation through NO production. Our findings suggest that the combined treatment of HT with CORM-2 is a potentially promising therapeutic strategy for treating endothelial dysfunction leading to atherosclerosis.
